# Supplementary material for: Acceptance and compliance with micronutrient powder (MNP) among children aged 6–23 months in northern Nigeria
Source: PLOS Glob Public Health. 2022 Oct 17;2(10):e0000961. doi: 10.1371/journal.pgph.0000961 (PMC10022258; doi:10.1371/journal.pgph.0000961)
Supplement: S1 Dataset — (ZIP) [file pgph.0000961.s008.zip › S8_Dataset/In-depth Interviews.zip!/In-depth Interviews/030715_K_BK_TA_2_CG.pdf]

A PHASE TWO (2) FEEDING TRIAL IN-DEPTH INTERVIEW WITH A FEMALE CAREGIVER AT YAMAMA KETERE, BIRNIN KEBBI LOCAL GOVERNMENT AREA, KEBBI STATE
CONDUCTED BY AMINA DUNIYA AND TRANSCRIBED BY TIMOTHY AUTA ON 3RD JULY, 2015

I: Thank you for the opportunity to discuss with you. Could you please tell me how many you are in the family?

R: We are many in this house. Mothers, in-laws and all

I: Like how many?

R: At the moment, the ones we are together with, including children we are four. The other wives are all in the farm house.

I: What about children between the ages of 6-23 months, how many are they?

R: Children?

I: Yes.

R: It is the one on my back that's six months.

I: From 6-23 months.

R: 6-23 months?

I: Yes

R: There is one with one year two years, 2 -3 years, 4-5 months; he is there with his mum. Then this girl, who is ten months.

I: We have heard of food challenges for young children in this setting related to the seasons. What mechanisms could be employed to cope these food challenges?

R: We don't have such challenges here. We cultivate our millets and sorghum, and even doing dry season, we still have them available.

I: I want to know about the seasonal foods. How to cope without them?

R: We feed the child with what is available like kunu, tuwo and even cook indomie for them.

I: Ok. Thank you. One thing we heard in other interviews that caregivers often give their children 6-23 months "teat". Could you please define or explain "tea"?

R: What do you mean by that?

I: Shayi

R: Ok. We give them.

I: This shayi (tea), what and what are the ingredients for shayi and how is it prepared?

R: We prepare it using lipton, sugar and other ingredients.

I: You said lipton, sugar and other things. What are the other things?

R: (She laughs) we don't prepare ours like yours. We don't use all the ingredients you use. We take it after breaking our fast in this Ramadan period and give the children also.

I: Ok. What and what do you use in the tea?

R: When we add lipton to hot water, we mix it with milk and sugar.

I: You said you add lipton, milk and sugar to make tea?

R: Yes

I: Thank you. This tea, is it with cold or hot water you prepare it?

R: We use hot water, by boiling. We get water from the well or borehole and boil it to prepare tea.

I: Thank you. Now, I want us to talk about the MNP that you received. Could you tell me about your experience with the product?

R: It makes her strong like that. Because before, the girl was not walking but now she has started crawling. She even attempts to push the door at the other end. She was weak before. Since I started giving her the MNP, she is now getting better.

I: You know I told you earlier that I am from Kaduna. What is fafakowa?

R: Trying to stand up as a young child, learning how to walk.

I: Ok. Thank you. Talk about any positive aspects of the experience with MNP.

R: It is making her body to be stronger. Yes, it gives her strength.

I: Thank you so much. Talk about any general challenges you had during the MNP trial?

R: Like fever, vomiting or dysentery?

I: Yes

R: Ok. She has not vomited or dysentery. Even the fever, if the body becomes hot, it easily goes down. It makes her to eat a lot.

I: When you want to open the sachet, do you experience any difficulty?

R: Sometimes, when opening, it is difficult, most especially when my hands are cold.

I: (Interrupted) so what do you do when opening?

R: He said we don't use mouth, sickle or knife to tear it open. I have to keep struggling to open it with my hand, until I succeed and pour it in her food..

I: Some caregivers mentioned that it was hard to store the MNP without it being taken by others or eaten by rodents. What was your experience?

R: No one of mine has been taken or eaten by the rodents. I store mine very well. I give it to her every day. Can it be given with cold kunu?

I: Pardon?

R: Can it be given with coold kunu?

I: No. Not with cold kunu, but warm one.

R: So the child cannot be given with stale kunu?

I: You can give the child but you have to warm the kunu.

R: Ok.

I: So, how do you give her?

R: I fetch little quantity of the kunu and mix it with the MNP. When she finish the mixed one, I give her more of the other one (the one not mixed with MNP). Because I have been told we shouldn't allow leftover. But there is a question. If I mix it and the baby doesn't finish all and I cover it and keep it somewhere, will it be effective if I give it to the baby later?

I: I will respond to all your questions later. Let's continue.

R: Ok.

I: The kunu you used in mixing the MNP, is it the cold one?

R: No. It is warm. Even when I have prepared that of others and she needs to take her MNP, I have to prepare little warm one for her specially. Just as you have said earlier, it is only given with warm foods. I don't accept giving her cold kunu.

I: Ok. Thanks so much. Some mothers indicated that it might be hard to remember to use MNP. Did you have similar experience and what did you do to remember?

R: (Interrupted). I have never forgotten to give her, I always remember. Even today, though I have not given her, I am just trying to finish cleaning the house before I prepare food for her and give her the MNP.

I: OK. How do you always remember? What solutions would you suggest to address each of the above mentioned barrier or challenge?

R: Why I always remember is because I have been told it is very good for the baby's health. I want it to benefit the child. For those who always forget, they are not good mothers. Because what so ever will benefit your child one doesn't suppose to forget

I: Thank you so much. But if you want to advice them, what will you tell them?

R: (She laughs) they should be determined to give it always. Why they don't always remember to give the MNP may be because they don't know the benefits to their children. If they know, they won't forget.

I: Ok. Thank you. Now, let's talk about how she takes the MNP. I earlier asked how you give her the MNP.

R: (She interrupted) I will add it to a spoon of food and give it to her. When she finish it and ask for more, I will give her more. But when she doesn't ask for more, I don't give her, until she ask. I make sure she finsih everything as you people instructed us.

I: So, what time do you give her the MNP? Morning/afternoon or night?

R: I give her in the morning. When I am busy, just like today that i am sweeping, I may give her around 12 noon. But I have never exceeded 2pm without giving her. Because my husband has instructed me to always give her iin the morning. He prefers morning to evening, to avoid not remembering.

I: Thank so much. Since you started giving the MNP. Have you observed whther she likes or dislikes it?

R: She doesn't reject it. When I give her she takes.

I: She doesn't notice whether something has been added to it?

R: Even if she noticed, she doesn't reject it, and she takes.

I: Thank you. You said earlier that it has increased her food consumption.

R: Yes. It also makes her stronger.

I: What type of food does she eat more now?

R: All varieties of food. Foods like tuwo, kunu, all she takes.

I: What about breastmilk?

R: She has been weaned because of the fasting period.

I: Ok. In other communities, we noticed that sharing of the MNP occurred.

R: (She interrupted) I didn't share it. I didn't share it with anybody. Like today, it is about .... twelve days and I make sure she takes every day.

I: So you didn't share it with any other child?

R: I didn't give anybody.

I: When you have leftover, what do you do with it?

R: I throw it away, since it is not to be given later.

I: Ok. Thank you. I now understand. You have been given sixty sachets, do you think the sixty will be enough for the sixty days?

R: Yes

I: You know you suppose to give one per day?

R: Yes

I: Will one be enough for the child per day?

R: Yes. Don't you see that is one per day that was instructed?

I: Yes. That's why i want to have your opinion.

R: I have never used more than one per day.

I: Do you think one per day is enough?

R: I have been told it is one I should give her and it's the one I have been giving her. Can I increase the number?

I: Not that you can increase. We want to know whether caregivers think it should be increased.

R: Caregivers? Like us or others?

I: Yes

R: It is what you tell us that we will follow. We have to follow your instruction. It is one you ask us to give and it is the one we will give. If we were told to give them at will, we would have been giving them even two sachets per day. But we won't give them because we don't know the effects it may have if we give not according to your instruction.

I: Ok. Thanks so much. Now, I want to know whether you see this product as a food,a medicine, or something else?

R: The person who gave me told me it helps the children's health and in brain formation, body building and make them to eat well. That it doesn't cause any vomiting. that is what he

told me and that's why i take it serious and always remember to give her. Insha Allah, I will give her for the sixty days.

I: Ok. That's why I say how do you see it? Is it a medicine or food?

R: I see it to be helping. Since it is said to help the child's health and brain formation, you know is intelligence we seek for our children.

I: If you're asked whether it is food or medicine, what will you say?

R: (She laughed) I will say they gave them food. I will call it food. Because we hear that it is milk against tamuwa.

I: Would you purchase this product in the market if it is sold cheaply?

R: I don't know since we don't know anything about it. If not because it was brought to us, we wouldn't have known about it. There are many other communities that don't know about it. That's why I take it with all seriousness. That's why we are also giving our children to see how it will benefit them.

I: That's why I am asking, would you purchase if these ones you have finish?

R: Yes, I will buy.

I: Ok. How much would you be willing to pay?

R: I cannot say a price since I don't know much about it.

I: Ok. What channels would you prefer for the distribution of the product?

R: For everyone to get enough in this community, you need to bring so many to go round, more than what you brought last time.

I: Through which channel?

R: Through the community leader.

I: What people most influence your opinions and knowledge about feeding practices?

R: The whole of us here respect and take instructions from our community leader. He is the only person we all trust.

I: Thanks. Who influenced your decisions regarding feeding your baby the MNP?

R: Old woman (my mother) influenced me.

I: Where would be good places to disseminate health information related to MNP?

R: Community leader's place or the mothers who are already trying the product.

I: What forms of media do you prefer for receiving MNP messages? Radio?

R: Yes. If there is any information or message passed through the radio, we use to get it.

I: What about poster/brochure/flyer?

R: Yes. It will be good, if there are people to explain to us that cannot read.

I: If another woman should ask you on how to use the product, what would you say?

R: I will explain to her how to use it. I will tell her to give the baby the MNP in the morning always to avoid forgetting about it. I will tell her to mix it with kunu and give it with the child. If she has leftover, she can drink the leftover. But if the child asks for more, she can give the child more of the kunu without the MNP in it.

I: What would you tell her to ensure she uses it but limit sharing?

R: I will tell her not to give anyone out of the ones she has been given.

I: Thank you so much. Finally, is there anything else that you can share about your experience with this product?

R: Honestly, I can't say anything on that now since it's not long I started using it. As you can see, I have not even finish one pack. Whether it is working as expected or not, I cannot tell for now.

I: Thank you so much. Do you have anything else to recommend to develop an effective program?

R: There is no recommendation. It is ok. Even as it is now, it is ok.

I: Ok. Thank you so much.
